# Comparing the performance of the public, social security and private health subsystems in Argentina by core dimensions of primary health care

**DOI:** 10.1093/fampra/cmw043

**Published:** 2016-07-02

**Authors:** Natalia Yavich, Ernesto Pablo Báscolo, Jeannie Haggerty

**Affiliations:** 1 National Scientific and Technical Research Council (Consejo Nacional de Investigaciones Científicas y Técnicas, CONICET), Center for Interdisciplinary Studies (Centro de Estudios Interdisciplinarios), National University of Rosario (Universidad Nacional de Rosario), Rosario, Argentina; 2 Department of Family Medicine, St. Mary’s Hospital Research Centre, McGill University, Montréal, Quebec, Canada

**Keywords:** Health Care Quality, Access, and Evaluation, health services research, integrated health care systems, Latin America, primary health care, public health

## Abstract

**Background:**

Most Latin American health systems are comprised of public (PubS), social security (SSS) and private (PrS) subsystems. These subsystems coexist, causing health care fragmentation and population segmentation.

**Objective:**

To estimate the extent of subsystem cross-coverage in a geographically bounded population (Rosario city) and to compare the subsystems’ performance on primary health care (PHC) dimensions.

**Methods:**

Through a cross-sectional, interviewer-administered survey to a representative sample (*n* = 822) of the Rosario population, we measured the percentage of cross-coverage (people with usual source of care in one subsystem but also covered by another subsystem) and the health services’ performance by core PHC dimensions, as reported by each subsystem’s usual users. We compared the subsystems’ performance using chi-square analysis and one-way analysis of variance testing. We analyzed whether the observed differences were coherent with the predominant institutional and organizational features of each subsystem.

**Results:**

Overall, 39.3% of the population was affiliated with the PubS, 44.8% with the SSS and 15.9% with the PrS. Cross-coverage was reported by 40.6% of respondents. The performance of the PubS was weak on accessibility but strong on person-and-community-oriented care, the opposite of the PrS. The SSS combined the strengths of the other two subsystems.

**Conclusion:**

Rosario’s health system has a high percentage of cross-coverage, contributing to issues of fragmentation, segmentation, financial inequity and inefficiency. The overall performance of the SSS was better than that of the PrS and PubS, though each subsystem had a particular performance pattern with areas of strength and weakness that were consistent with their institutional and organizational profiles.

## Introduction

Most Latin American ‘health systems’ are composed of public (PubS), social security (SSS) and private (PrS) subsystems. Each has its own philosophical underpinning, funding base, management framework and intended catchment population. Though the performance of their respective health services is expected to vary depending on the population served and each subsystem’s organizational and institutional features (1), an oversimplified portrait persists in policy documents and some of the peer-reviewed literature. This portrait characterizes the private subsystem as more efficient, accountable and quality driven than the public health subsystem, in turn biasing public opinion and policy debate (2).

This oversimplified portrait masks the differences within each of the subsystems and the fragmented nature of the available data does not permit a direct comparison between subsystems. In fact, the so-called private subsystem is actually a collection of different governance structures and delivery systems of varying degrees of complexity. The SSS can also include multiple governance organizations and delivery institutions. Even the PubS can have different levels of municipal, state or national government health care schemes. Furthermore, the population can be covered by different subsystems, with the PubS serving as the ultimate safety net for all. Cross-coverage of the population, interdependence between subsystems and the changes that have occurred in the Latin American private health market during recent decades require a more nuanced analysis (3).

Across Latin America, countries are making efforts to move toward the development of integrated, primary health care (PHC)-based health systems to improve access, efficiency, equity and quality for the whole population (4,5). Our purpose is to compare the performance of each subsystem using a common set of tools and to understand the degree of overlap between subsystems in order to help governments to focus strategies to improve governance and delivery (6).

This study is guided by the World Health Organization (WHO) framework for health system performance (1,7). According to this framework, health systems are characterized by key functions such as stewardship, financing and resource generation, which translate into service provision to the population. High-performing services have instrumental goals of providing high quality, responsive and affordable care. This care is the means to achieving the fundamental goals of the health system: to improve the average health status of the population (including reducing health inequalities); to be responsive to the non-medical expectations of the population (respect for people and a client orientation); and to ensure fairness in financial contributions. PHC services contribute to these goals through their instrumental goals of timely access, comprehensiveness, continuity of care, person-and-community orientation and technical quality of care.

The premise of this study is that, in a context of multiple health subsystems, differences in the key functions of each system will translate into differences in the extent to which PHC services achieve their instrumental goals in core PHC dimensions. Our research questions are two. Given the fragmented nature of the Argentinean health system, what is the extent of cross-coverage between subsystems? How well is each subsystem performing on core PHC dimension, as reported by their regular users? We hypothesize that (i) the emerging patterns of strengths and weaknesses in the performance of each subsystem will be consistent with its funding, governance, and organizational particularities and (ii) there will be a high rate of cross-coverage largely due to the strategies used by the population to complement or avoid the limitations of the subsystem with which they were originally affiliated.

The objective of this study is to estimate the extent of subsystem cross-coverage in a population in a given geographic area and to compare the subsystems with respect to their performance on PHC core dimensions as reported by the affiliated population. We use the municipality of Rosario, Argentina, as the study case.

## Rosario health system

Rosario is the third most populous city in Argentina (948312 inhabitants). Argentina is a federal democracy, with 23 provinces and the Autonomous City of Buenos Aires. Funding and delivery of public services are the authority and responsibility of each jurisdiction. The regulatory frameworks share common features across jurisdictions, but health services delivery and organization vary. Depending on fiscal capacity, funding and delivery has been further decentralized to the municipal level. Like many Latin American countries and jurisdictions, Rosario’s population is covered by segmented public, social security and private systems. It shares most of the institutional and organizational features of the Latin American segmented health systems, synthesized in Table 1. Below, we describe Rosario’s subsystems, including features that are distinct from the typical Latin American case.

**Table 1.** Typical institutional and organizational features of Latin American health subsystems

Latin American subsystems	Public	Social security	Private
Funding	General taxes	Employers’ and employees’ payroll deductions	Clients’ voluntary contributions (prepaid insurance and direct payment)
Population served	Population without contribution capacity	Workers in the formal labour market	High-, middle- and low-income populations
Health services coverage	Basket of basic free services. Other services may require out-of-pocket payments or may not be offered	Broader basket of services compared to the PubS, but still requires copayments	For high-income population, a broad basket of services is offered at a high cost
			For low- and middle-income population (uninsured), complementary or supplementary plans are offered at a relatively high cost
Authorities/management	National, subnational and local ministries/secretaries	Single or multiple social security organizations	Multiple for-profit companies and non-profit organizations
Integration of funding and services provision	Vertical integration: owned health services	Vertical integration: owned health services	Contractual integration: purchase health services through per capita and/or fee-for-services contacts
Main professionals’ remuneration system	Salaries	Salaries	Fee-for-service

### Rosario’s public subsystem

Rosario is one of the country’s richest municipalities, although ~115000 of its residents live in slums (8). Since the 1990s, municipal authorities have invested substantially to develop a publicly funded local health system (PubS) that currently provides a wide range of free health care services, aimed mainly at the population that is not covered by other systems—approximately a third of the city’s population. Rosario’s PubS is a vertically integrated system of ambulatory health centres (with multidisciplinary teams), hospitals, an ambulatory medical specialities centre, a rehabilitation centre and an emergency system. Resources are assigned to the system based on public budgets. Per capita health spending in the Rosario PubS was $1907 Argentinean pesos [$479 US dollar (USD), according to the official exchange rate at 12 December 2010] in 2010 (9,10).

Furthermore, health professionals have intentionally developed Rosario as a model of participatory management with a multidisciplinary first level of care that has a strong role in coordinating the system. Rosario has been recognized in Latin America as a PHC champion and an example of a PHC-based health system because of the development and achievements of its PubS (11).

### Rosario’s social security subsystem

The SSS is an employment-based insurance, with financing based on a percentage of workers’ salaries and not on premiums that can be adjusted. The Argentinean Ministry of Health regulatory framework requires that SSS insurers provide an obligatory broad basket of services to workers and their families. The Rosario SSS subsystem is made up of around 50 of the nearly 300 entities functioning around the country. The entities vary in scope and size and have different models of care and management, all of which together attempt to provide a broad package of services within the limited financing available from worker contributions. Unlike other Latin American SSS entities, in Argentina and Rosario few entities have their own health services. Instead, they purchase health services through per capita and/or fee-for-services contracts with private providers through selective commissioning. They use different strategies to control, monitor and improve health care quality such as economic incentives, care guidelines and the development of information and auditing systems (12). Per capita health spending in the SSS ($637 USD) is 33% greater than in the PubS, but 37% lower than for private insurers (10).

### Rosario’s private subsystem

The PrS comprises over 30 companies. There are three types of entities: voluntary prepaid insurance companies that offer a variety of premiums; emergency services companies that provide emergency care and urgent home care; and social services companies that offer a basic basket of essential ambulatory services through cheap premiums. Like the SSS, PrS entities contract service delivery from private providers and professional associations. Clients choose their own providers from the range of entities and professionals offered by their insurer. Services are provided in free-standing health practitioner offices, independent clinics and/or clinics owned by insurance companies. Clinics tend to be organized by discipline, so multidisciplinary work is uncommon. Most health professionals work under an entrepreneur model and with fee-for-service remuneration depending on the number and type of medical services. In contrast to the SSS, the PrS has a more passive role in controlling and coordinating health services, with minimal mechanisms to regulate clients’ use of services and health professionals’ practices (12). Per capita health spending in the PrS ($873 UDS) is 82% greater than in the PubS and 37% higher than in the SSS (10).

In this study, we selected a representative sample of the population of Rosario to assess the subsystem to which individuals are predominantly affiliated and reported each subsystem’s performance on core dimensions of PHC as reported by each subsystem’s usual users. We then compared the performance of each of the subsystems to discern whether the observed differences are consistent with the predominant institutional and organizational features of the subsystems.

## Methods

### Study design, sample and data collection

We conducted a cross-sectional interviewer-administered survey using a multistage, stratified sampling strategy designed to obtain a sample representative of the age/sex and socio-economic strata of the municipality of Rosario. The initial sampling unit was the census tract stratified by the proportion of households below the poverty threshold. Once the census tract was selected, all residential dwellings in the city blocks that were located within the perimeter of the census tract were contacted. In active households, all members were enumerated and one respondent was randomly selected until the required sample size in each age strata was obtained (age < 15 years, 15–49 years and 50+ years). The final sample of 822 residents was designed to represent the age–sex demographic strata, with a 95% margin of error of 3.5%. The survey was conducted between December 2010 and January 2011.

We assigned respondents to a particular subsystem based on their affiliation to a specific facility that they reported as their regular or most frequently used place of medical care (from now on, referred to as usual source of care). As some facilities might provide health care for people affiliated with different subsystems, the respondents were asked to indicate the type of coverage used to receive medical care at that facility. We did not assign respondents based on their health coverage status because it is likely to find people who are affiliated with one subsystem and also covered by another subsystem (e.g. covered by the PrS and the SSS but affiliated with the SSS).

Those without a usual source of care (*n* = 14) were assigned to the subsystem where they had their last visit. We excluded 21 people who reported affiliation to either non-governmental health organizations or traditional medicine, since these were beyond the scope of this study.

### Survey instrument

The questionnaire included 67 questions related to seven sections: (i) demographic and socio-economic conditions of the respondent and his/her family, with questions on health insurance, employment, literacy and housing; (ii) access to health care within populations with chronic conditions, where respondents were asked to indicate whether they had been diagnosed with any of a number of conditions and if they were currently receiving treatment for those conditions; (iii) health care needs, with questions about their experiences in the last 12 months; (iv) continuity of care, where respondents were asked to identify their usual source of care and respond to a number of questions about relational and informational continuity of care at the usual source of care, all based on their experience in the last 12 months; (v) person-and-community-oriented care, again with a focus on the usual source of care during the last 12 months; (vi) accommodation, which included two sets of questions related to the respondents’ experience with their usual source of care, one set based on their experience during the last 12 months and the other based on their experience during the last consultation; and (vii) Health care use, including questions on health services utilization during the last 12 months and the last medical consultation.

The questions for respondents younger than 15 years of age were answered by the adult with the greatest responsibility for their care. The questions were mostly closed, with a mix of reporting and evaluative response options, and were taken as much as possible from a Canadian validated instrument (13) and other previous surveys conducted in Argentina (11,12).

### Analytic strategy

Analysis was conducted in two stages: (i) reduction of variables into underlying dimensions of PHC performance and (ii) comparison of subsystems by variable and dimension.

### Dimensions of primary health care performance

We reduced the large number of reported variables into broad dimensions of PHC using a categorical nonlinear principal components analysis (CATPCA) (14). CATPCA has the same objectives as traditional principal components analysis, but it allows for variables with mixed measurement levels, transforming them into categorical variables and creating linear combinations between the transformed variables.

The relationship between the components and variable loadings can be represented graphically through component loading plots of the principal component space, where the axes are the principal components. In these plots, variables with relatively long vectors fit well into the solution, with the squared length of the loading vector equalling the variance accounted for. When vectors are long, the cosines of the angles between the vectors indicate the Pearson’s correlation between the quantified variables. The slope of the vectors indicates the relationship of each variable with each component. An angle of 90° indicates no correlation between variables, 180° indicates negative correlation and 0° indicates positive correlation (14). All analyses were conducted using SPSS version 21.

We hypothesized *a priori* which variables best represented each dimension and sub-dimension. We used 19 variables: 12 ordinal, 6 nominal and 1 continuous. Two components accounted for >30% of the variance in each subsystem (PubS 31.8%; SSS 32.2%; PrS 31.0%), with Cronbach’s alphas of ~0.90 (PubS 0.899; SSS 0.888; PrS 0.912).

### Comparison of subsystems

Subsystems were compared to look for significant differences in the demographic and socio-economic characteristics of the respondents in each system, using chi-square analysis for categorical variables and one-way analysis of variance tests for continuous variables. We conducted a similar analysis for each of the variables for the following PHC performance dimensions: access to health care services; continuity of care; person-and-community-oriented care and health services utilization. According to the WHO framework for health system performance (1,7), high performance on these dimensions is a means to achieve the health system’s fundamental goals, particularly the goal of being responsive to the non-medical expectations of the population.

Given that there is no consensus on performance standards to determine whether a specific benchmark was achieved or surpassed, we described performance achievements in relative terms as per Murray and Frenk (1). As in other studies, we used a combination of statistical significance and judgment to denote ‘weak’ or ‘strong’ performance (2,6). For each variable that was statistically significantly different by subsystem, we used our judgment to denote the ‘strong’ or ‘weak’ subsystem(s) relative to the other(s). For each PHC dimension and sub-dimension, we identified as ‘strong’ the subsystem with the strongest variables and as ‘weak’ the subsystem with the weakest variables.

## Results

### Demographics and socio-economic characteristics by health subsystems

Overall, 39.3% of the population was affiliated with the PubS, 44.8% with the SSS and 15.9% with the PrS. We found significant differences among subsystems by participant sex and age and household socio-economic conditions, including the head of household’s employment status and educational level and the overall household living conditions (Table 2). The respondents affiliated with the PubS subsystem were more likely to be younger, male, unemployed, less educated and to report worse living conditions than respondents in the other subsystems. For respondents in both the SSS and PrS subsystems, the respondent profile was older, more female and educated, and among household heads, unemployment was lower and the percentage of retired workers was higher. The higher proportion of older adults in the SSS is expected, given that it includes the national insurance for seniors and pensioners.

**Table 2.** Study population demographics, socio-economic conditions and health insurance status by health subsystem, Rosario, 2011 (*n* = 801)

		Total		Public subsystem		Social security subsystem		Private subsystem		Significance level
		*n*	%	*n*	%	*n*	%	*n*	%	
Demographics										
Population		801	100	315	39.3	359	44.8	127	15.9	–
Sex	Male	418	52.2	182	57.8	179	49.9	57	44.9	0.024
Age	0–12 years	155	19.4	80	25.4	58	16.2	17	13.4	0.000
	13–20 years	120	15.0	68	21.6	37	10.3	15	11.8	
	21–60 years	358	44.7	149	47.3	145	40.4	64	50.4	
	61–99 years	168	21.0	18	5.7	119	33.1	31	24.4	
Socio-economic conditions										
Household head, employment condition	Employed	635	79.4	277	87.9	262	73.2	96	75.6	0.000
	Unemployed	17	2.1	15	4.8	1	0.3	1	0.8	
	Retired	148	18.5	23	7.3	95	26.5	30	23.6	
Household head, educational level	Never attended school or incomplete primary	131	16.4	51	16.2	57	15.9	23	18.1	0.002
	Complete primary	209	26.1	104	33.0	80	22.3	25	19.7	
	Incomplete secondary	138	17.3	62	19.7	54	15.1	22	17.3	
	Complete secondary	207	25.9	73	23.2	99	27.7	35	27.6	
	Incomplete or complete post-secondary or university	115	14.4	25	7.9	68	19.6	22	17.3	
Type of housing^a^	Deficient	334	41.7	172	54.6	114	31.8	48	37.8	0.000
Health coverage										
Cross-coverage between subsystems^b^	No cross-coverage	426	53.2	201	63.8	164	45.7	61	48.0	–
	Cross-coverage	325	40.6	114	36.2	179	49.9	32	25.2	
	Lost insurance given by the subsystem of affiliation during the last year^c^	50	6.2	–	–	16	4.5	34	26.8	

^a^A house is considered deficient when it has at least one of the following characteristics: (i) floor—dirt or loose brick, (ii) walls—wood, metal or fibrocement sheets, (iii) walls without external plaster or coating, (iv) roof without internal coating or ceiling only or (v) has a water source outside of the home.

^b^The subsystem affiliation refers to the subsystem of the provider reported as the usual or most frequently used place of medical care. The term coverage includes two situations: people with insurance from the SSS or the PrS and people with a factual link to the PubS, as the PubS does not provide insurance to anyone and theoretically provides coverage to everyone.

^c^Shifted from SSS to PrS, vice versa or lost insurance.

Cross-coverage (usual care from one subsystem but also covered by another subsystem) was reported by 40.6% of respondents. Half of those affiliated with the SSS had complementary or supplementary PrS coverage. Over a third of PubS affiliates had SSS and/or PrS coverage and a quarter of PrS affiliates had SSS coverage.

### Relationship between components by system

In all three subsystems, the first component combines the dimensions we hypothesized as continuity of care, person-and-community-oriented care and health utilization while the second maps onto accessibility. Health care access within populations with chronic conditions maps onto the first component in both the SSS and PrS and onto the second component in the PubS. Except for question 13 in the SSS, unmet health care needs maps onto the first component.

**Figure 1.**
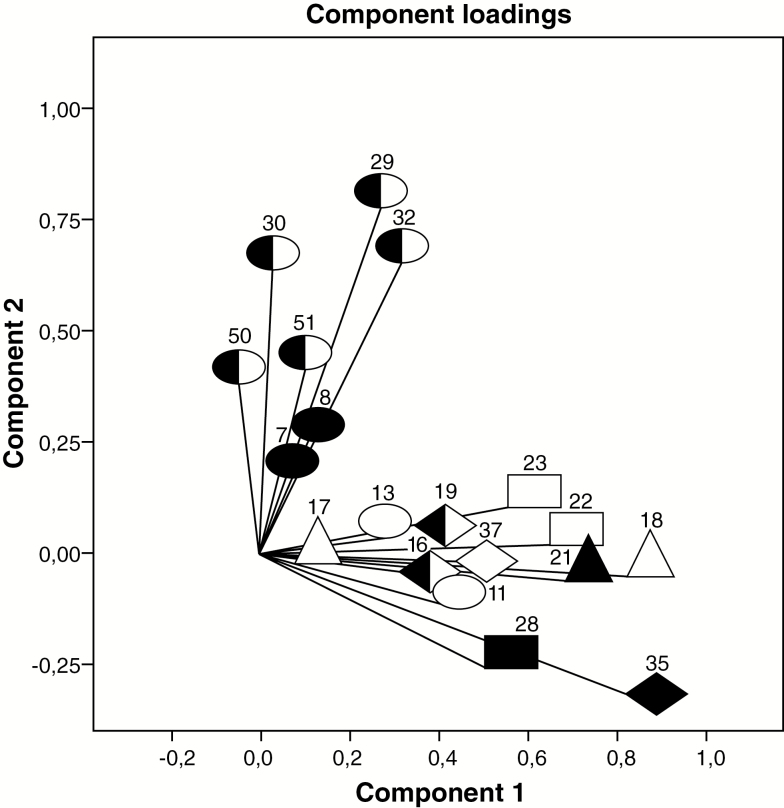
Public subsystem: component loadings from categorical non-linear principal components analysis on 19 primary health care performance variables.

**Figure 2.**
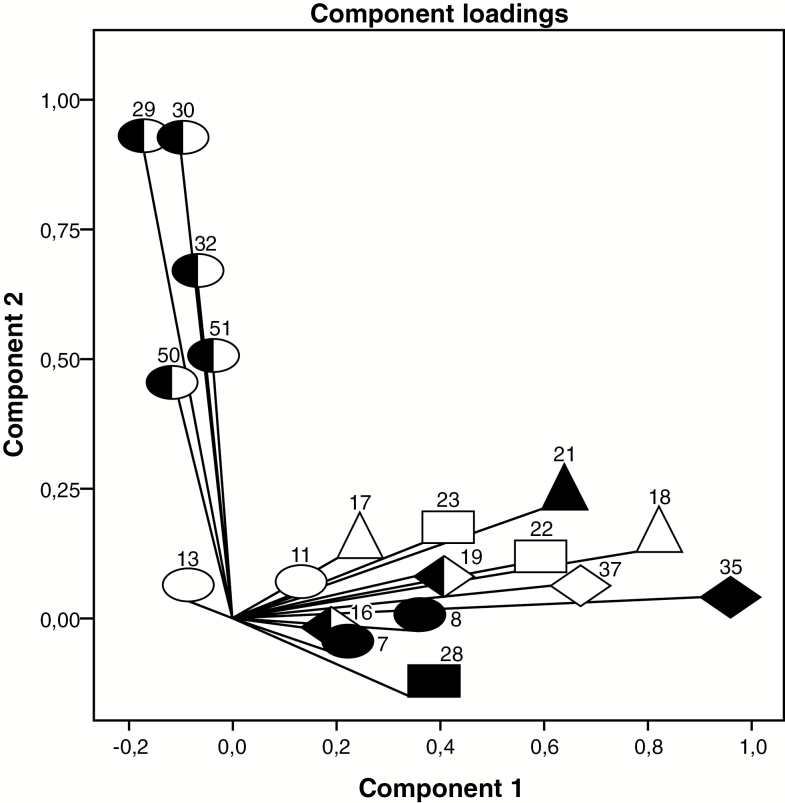
Social security subsystem: component loadings from categorical non-linear principal components analysis on 19 primary health care performance variables. For explanations on symbols, please see Figure 1.

**Figure 3.**
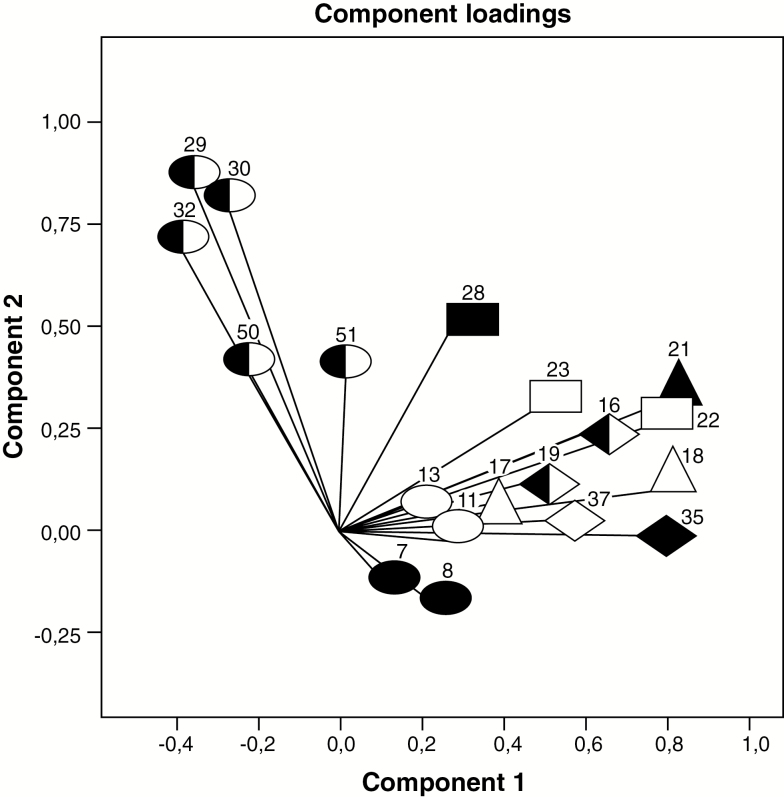
Private subsystem: component loadings from categorical non-linear principal components analysis on 19 primary health care performance variables. For explanations on symbols, please see Figure 1.

The CATPCA component loading plot showed a positive correlation between components in the PubS (Fig. 1); a null correlation in the SSS (Fig. 2); and a negative correlation in the PrS (Fig. 3).

### Primary health care performance by health subsystems

Subsystems showed significant differences in performance for all PHC dimensions and sub-dimensions, with the exception of unmet health care needs and medical consultation rates (Table 3).

**Table 3.** Differences in variables that measure the primary health care dimensions and sub-dimensions among the user populations of different health subsystems

Variables, by primary health care dimensions^a^ and sub-dimensions			Public subsystem		Social security subsystem		Private subsystem		Significance level
			*n*	%	*n*	%	*n*	%	
Access to health care services									
Unmet health care needs	Required health care during the last 12 months		263	83.5	311	86.6	106	83.5	0.466
	Sought out health care each time he/she felt a health care need during the last 12 months (Q11)^b^		239	91.2	294	94.5	100	94.3	0.209
	Abandoned health care seeking during the last 12 months (Q13)		19	7.3	14	4.5	5	4.8	0.327
Access to health care in populations with chronic conditions	Has been diagnosed with a chronic condition (Q7)		64	20.3	117	**32.6**	35	**27.6**	0.002
	Currently receiving treatment for chronic conditions (Q8)		49	76.9	111	**94.9**	30	**86.1**	0.002
Accommodation/ accessibility	Level of comfort with appointment system of his/her USC during the last 12 months (Q29)	*Not at all or only somewhat comfortable*	97	32.8	47	13.5	10	8.1	0.000
		*Moderately comfortable*	71	24.0	56	16.1	10	8.1	
		*Very or completely comfortable*	128	43.2	244	**70.3**	103	**83.7**	
	Possibility of obtaining appointment in his/her USC by phone during the last 12 months (Q30)	*Not at all or only somewhat possible*	210	77.5	48	14.1	7	5.8	0.000
		*Moderately possible*	18	6.6	36	10.6	12	10.0	
		*Very or completely possible*	43	15.9	257	**75.4**	101	**84.2**	
	Ease of obtaining an appointment for an acute problem in his/her USC during the last 12 months (Q32)	*Not at all or only somewhat easy*	54	20.1	33	11.4	18	15.1	0.000
		*Moderately easy*	105	39.0	100	34.6	31	26.1	
		*Very or completely easy*	110	40.9	156	**54.0**	70	**58.8**	
	Ease of obtaining an appointment for a routine check-up in his/her USC during the last 12 months	*Not at all or only somewhat easy*	34	12.8	26	8.5	7	7.3	0.000
		*Moderately easy*	103	38.7	91	29.6	26	27.1	
		*Very or completely easy*	129	48.5	190	**61.9**	63	**65.6**	
	Number of days between the request for an acute problem and the visit, considering the last visit in his/her USMC	*>week*	30	27.3	25	22.3	4	8.9	0.042
		*2–7 days*	35	31.8	44	39.3	13	28.9	
		*Next day or same day*	45	40.9	43	38.4	28	**62.2**	
	Time invested to obtain the last visit appointment in his/her USC among those who had to request one in person (Q50)	*>1 hour*	19	10.4	9	3.7	3	3.9	*0.000*
		*30 minutes to 1 hour*	39	21.4	26	10.6	6	7.8	
		*<30 minutes or by phone*	124	68.1	211	**85.8**	68	**88.3**	
	Time spent in the waiting room prior to receiving health care during the last visit at his/her USC (Q51)	*≥1 hour*	55	21.2	40	12.9	14	14.0	*0.014*
		*30 minutes < 1 hour*	64	24.6	56	18.0	22	22.0	
		*<30 minutes*	141	54.2	215	**69.1**	64	**64.0**	
Number of variables for which performance is high (over the mean) within those variables with significant differences			0/9		8/9		9/9		9
Continuity of care									
Relational continuity	Currently has a USC, a place that she/he regularly or frequently uses each time that she/he needs medical care (affiliation)		303	**96.2**	352	**98.1**	118	92.9	*0.024*
	Has been seeking care at the USC for a year or more (Q17)		271	88.3	321	90.7	109	86.5	*0.155*
	Is currently a regular patient of a particular physician at the USC (Q18)		195	**63.5**	230	**65.0**	61	48.4	*0.003*
Informational continuity based on last 12-month experience	How complete is her/his medical record at the USC (Q21)	*Not at all or only somewhat complete*	15	6.3	19	6.4	12	13.8	*0.004*
		*Moderately complete*	33	13.9	54	18.3	10	11.5	
		*Very or totally complete*	190	**79.8**	222	75.3	65	74.7	
Number of variables for which performance is high (over the mean) within those variables with significant differences			3/3		2/3		0/3		3
Person-and-community-oriented care									
Whole-person care based on last 12 months experience	How well do the staff know her/ him at the USC (Q22)	*Not at all or only somewhat well*	142	46.3	180	50.8	72	57.1	*0.013*
		*Moderately well*	59	19.2	81	22.9	26	20.6	
		*Very or completely well*	106	**34.5**	93	26.3	28	22.2	
	How possible would it be for the USC to contact her/him to go to her/his appointments (Q23)	*Not at all or only somewhat* possible	187	60.9	260	73.4	99	78.6	*0.000*
		*Moderately* possible	53	17.3	57	16.1	18	14.3	
		*Very or completely possible*	67	**21.8**	37	10.5	9	7.1	
Population orientation based on last 12 months experience	Which phrase best describes your USC: it takes care of…	*Visits or visits and patients*	171	55.7	274	77.4	101	80.2	*0.000*
		*Patients and their families or patients and their families and communities*	136	**44.3**	80	22.6	25	19.8	
	Participated in a community activity organized by the USC during the last 12 moths		25	**8.1**	4	1.1	2	1.6	*0.000*
	How active the USC has been in seeking out solutions to community problems (Q28)	*Not at all or only somewhat active*	382	69.0	539	77.2	195	79.3	*0.000*
		*Moderately active*	27	4.9	28	4.0	15	6.1	
		*Very or completely active*	145	**26.2**	131	18.8	36	14.6	
Number of variables for which performance is high (over the mean) within those variables with significant differences			5/5		0/5		0/5		5
Health services utilization									
Annual consultation rate for population aged 18–64 years (Q37)			3.1		2.9		3.1		*0.528*
Comprehensiveness	Health services used during the last 12 months (Q35)	*General physician or specialist*	146	55.9	115	36.5	47	44.8	*0.000*
		*General physician + specialist*	69	26.4	108	34.3	28	26.7	
		*At least general physician + specialist + dentist*	46	17.6	92	**29.2**	30	**28**.**6**	
Appropriateness of place and provider	Population whose USC is an *emergency or after-hour service* (Q16)		13	**4.2**	29	**8.2**	29	23.4	*0.000*
	Professional profile of her/his regular physician at her/his USC (Q19)	*General physician*	183	**93.3**	191	83.0	53	86.9	*0.006*
		*Specialist*	13	6.7	39	17.0	8	13.1	
Number of variables for which performance is high (over the mean) within those variables with significant differences			2/3		2/3		1/3		3

USC, usual source of care; USMC, usual source of medical care.

^a^Dimension definitions:

• Access to health care services: the ease with which the population is able to use appropriate services in proportion to their needs.

• Continuity of care: the degree to which a series of discrete health care events are experienced as coherent, connected and consistent with the patient’s medical needs and personal context.

• Person-and-community-oriented care: the extent to which primary care clinicians consider and respond to their patients’ physical, emotional and social needs considering their familiar and community context.

• Health services utilization: measures include type and degree of services used.

^b^The number of questions is to identify only those variables included in the CATCPA.

The main strengths of the PubS were continuity of care, appropriateness of place and provider and person-and-community-oriented care. The performance of the PubS in continuity of care and appropriateness of place and provider approximates the SSS, but it has a lower percentage of people whose usual source of care is an emergency or after-hour service, a higher percentage of people whose regular provider is a general physician (instead of a specialist) and a higher percentage of people who are more likely to have very or totally complete medical records in their usual source of care. The performance of the PubS for person-and-community-oriented care is far superior to that of the PrS and the SSS.

Accommodation, access to health care in populations with chronic conditions and comprehensive use of services are the major performance weaknesses in the PubS. The PubS had the weakest performance on the accessibility dimension, with statistically significant differences on all nine indicators and respondents expressing particular difficulty with the appointment process. Compared to the SSS and PrS, the percentage of people diagnosed with chronic conditions in the PubS is significantly lower, as is the percentage of people diagnosed with at least one chronic condition who are currently receiving treatment. It should be noted that in the PubS, health care access in the population with chronic conditions and accommodation are combined in component 2 (Fig. 1). Although there were not significant differences between subsystems, the PubS had the worst performance on unmet health care needs. Finally, affiliates in the PubS were least likely to consult a wide range of services (comprehensive access).

The PrS had a performance profile that was the opposite of the PubS. It had the weakest performance in appropriateness of place and provider, continuity and person-and-community-oriented care. The performance of the PrS for access to health care in populations with chronic conditions was better than that of the PubS, but the rate of chronic illness detection and treatment were 5.0 and 8.8 points lower, respectively, than those of the SSS. The performance of the PrS on comprehensiveness was almost as good as the SSS, and it had the strongest performance on all but one (question 51) of the nine variables related to accommodation. Despite the strong performance of the PrS, respondents in this subsystem were the most likely to have used the emergency room or after-hours service, as these are the usual source of care for part of this population.

The SSS was similar to the PrS on accessibility conditions and to the PubS on continuity of care, but it outperformed the other two subsystems on comprehensiveness, access to health care in populations with chronic conditions and unmet health care needs, though for the last variable, there were no significant differences between subsystems. The performance of the SSS in person-and-community-oriented care was better than that of the PrS but substantially lower than that of the PubS. Regarding appropriate use of place, the performance of the SSS was similar but inferior to that of the PubS. With respect to appropriate use of provider, the SSS had the highest proportion of population whose regular provider is a specialist, probably due to the higher proportion of senior population and the higher rate of chronic illness detection and treatment.

**Table:** 

Dimension	Sub-dimension	Shape	Question number
Access to health care services	Unmet health care needs	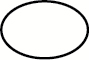	11 and 13
	Health care access in population with chronic conditions	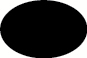	7 and 8
	Accommodation	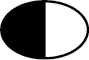	29, 30, 32, 50 and 51
Continuity of care	Continuity relational	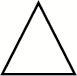	17 and 18
	Informational continuity	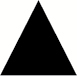	21
Person-and-community- oriented care	Whole-person care	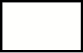	22, 23
	Population orientation	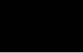	28
Health services utilization	Medical consultations	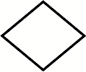	37
	Comprehensiveness	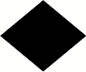	35
	Appropriateness of place and provider	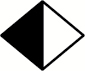	16 and 19

## Discussion

In this study of a geographically bounded population, we found cross-coverage by the different subsystems in a high proportion of respondents. When analysing respondents’ experiences with their affiliated subsystem or usual source of care, the performance of the PubS is weak on accessibility but strong on person-and-community-oriented care, the opposite of the PrS, which is strong on accessibility but weak on person-and-community-oriented care. The SSS combines the strengths of the other two subsystems and is particularly strong in continuity of care and detection and treatment of people with chronic conditions. In this section, we interpret the performance and the correlational structures between performance dimensions in light of each subsystem’s institutional and organizational arrangements.

The pitfall of accessibility to the PubS is due to the lack of incentives to attract users and the insufficient supply of services to meet user demands (6,15–17). For instance, the PubS regulates (or rations) the demand for services by requiring users to present requests for services in person rather than offering the possibility of making telephone appointments. This situation makes it difficult for certain populations to achieve a continuous affiliation and linkage to this subsystem (e.g. working age males, sick or disabled people) and may explain the low levels of detection and treatment of individuals with chronic conditions. The positive correlation between the CAPTCA components suggests that improvements in overall accessibility and in the identification and ongoing care of people with chronic conditions would have a positive effect on the overall direction and continuity of care and would lower emergency care usage, in turn resulting in more integrated use of the health system.

Although timely accessibility is problematic in the PubS, the annual consultation rate and unmet health care needs are not statistically significantly different from the other subsystems. This contrasts with other studies that show higher use rates in the PrS (2). Our findings may be not generalizable to other settings as Rosario’s PubS has been the focus of public policy and has experienced an extraordinary level of service development and expansion with free health care delivery. Though this is not an efficiency study, it should be noted that this level of heath service utilization is reached by the PubS, with significantly lower expenditure than the SSS or the PrS.

In contrast to other studies, we found that the PubS outperforms the PrS in health care orientation and continuity of care (6). Again, this likely reflects Rosario’s investment in multidisciplinary teams with a strong orientation to PHC principles and values (18). We anticipate that the PubS in other contexts, which have not benefited from orientation or investment similar to those in Rosario, will have even more problematic accessibility and fewer achievements in person-and-community-oriented care or health service utilization.

Our study confirms the findings from several studies that the strength of the PrS comes from its comparatively more agile appointment processes (2,6,19). To this, Rosario adds the provision of home-based care services for urgent care, and the wide supply and relatively unrestricted access to specialized care. These features are reinforced by the PrS’s market strategy to capture low-income clients who are unsatisfied with the PubS’s appointment conditions and middle- and high-income clients who are reluctant to use regulated SSS services. This strategy leads to two types of populations within the PrS: one made up of individuals from a middle-to-low socio-economic level who are covered by a reduced basket of essential services and/or emergency services (34.6% in our sample) and another composed of individuals of a middle-to-high socio-economic level who are covered by a comprehensive insurance (65.4% in our sample). This explains why the PrS had a higher percentage of people with low-quality living conditions than the SSS in our sample.

Our data show that a high proportion of middle- and low-income populations pays for private coverage in order to have easier access to urgent and emergency care. Though duplicate or supplementary coverage provides additional access options for affiliates of the PubS and SSS, it introduces more fragmentation into their health care process and more segmentation and financial inequity into the health system. It also increases health system costs and inefficiencies (3,4), since most SSS and PubS users covered by the PrS do not use their private insurance on a regular basis.

The emphasis of the PrS system on ease of access that is not supported by strong continuity of care or a person-and-community orientation disrupts the virtuous synergy between access and continuity of care and may compromise quality of care. The lack of mechanisms to regulate and monitor service use and to coordinate health care processes is associated with overprescription and compromised quality of technical care (6). The absence of health providers who take responsibility for and orient patients’ care processes fosters the observed pattern of consultations oriented toward acute care, with the highest use of emergency and specialist services, which generate higher costs and inefficiencies in the system.

The SSS’s strong performance in accessibility, continuity of care and detection and treatment of people with chronic conditions relates to a combination of market and regulation mechanisms. SSS entities purchase services using contractual mechanisms including economic incentives to promote timely access. Through auditing and gate-keeping functions, they promote continuity and appropriate use of services. The SSS’s even and strong performance is consistent with SSS entities’ use of different management strategies to improve access and quality of care. Despite these strengths, the SSS and PrS need to develop care that is more person-and-community oriented.

Lessons learned from the evidence indicate that the performance of the PrS could be improved by introducing incentives and/or new contractual modalities aimed at improving the continuity of care and increasing the efficiency of service use through the enhancement of its monitoring and information systems. Both the SSS and PrS would benefit from adopting a PHC focus, especially to improve person-and-community-oriented care. PubS accessibility conditions could be improved by the implementation of better appointment systems, including the option to schedule appointments by phone. Monitoring cross-coverage could help to track the flows of resources and patients between subsystems, justify and inform the implementation of mechanisms to compensate public expenditures in patients covered by the other subsystems, and develop mechanisms to reduce the fragmentation of the health care process and the segmentation of the system.

The major strength of this study is that it compares subsystem performance using common indicators based on respondents’ predominant subsystem affiliation. The level of cross-coverage between subsystems demonstrates the importance of basing performance on the subsystem affiliation rather than the client or enrolment list from each subsystem. However, there are important limitations. The indicators provide very little information on the technical quality or appropriateness of care. The judgement of stronger or weaker performance is determined by judgement about what is considered ‘better’ based on statistically significant differences and not on a widely recognized benchmark. Finally, the choice of Rosario as a case limits the generalizability of the findings to other low- and middle-income contexts in Latin America, including within Argentina. Rosario’s publicly funded system is an exemplar of a PHC-based and community-oriented system that has been the focus of public investment and social policy for decades. Although this case is not generalizable to other settings, the findings about system weaknesses and how they might be reinforced are transferable to other settings.

## Conclusions

We used a common set of tools in a geographically bounded population to compare health services performance in different subsystems in the Rosario health system. This allowed us to determine the percentage of cross-coverage between respondents, which is likely to be similar to other Argentinean settings; measure performance based on the experience of the subsystems’ regular users; and make direct comparisons regarding the performance of these health subsystems.

We found a high percentage of cross-coverage between subsystems, which is a marker of system fragmentation and segmentation and which may also indicate financial inequity and inefficiency. We found different performance patterns in each subsystem, consistent with the subsystems’ institutional and organizational profiles. Our findings allowed us to refute the claim that the PrS services perform better than those of the PubS or the SSS in any scenario. Our findings also suggest that the PrS is not more efficient than the PubS or the SSS, at least in the case being studied.

We showed that in Rosario, as in many Latin American countries, the private subsystem does not exclusively serve the high-income population, as often claimed (20), but that it is embedded in the middle- and low-income populations.

This study provides evidence on the performance of the PubS and the SSS, which is limited and poor quality in low- and middle-income countries, and on the performance of the PrS, which is scarce in all countries and particularly in middle- and low-income countries. It contributes to understanding the dynamics between subsystems, particularly through the determination and analysis of cross-coverage between subsystems. Based on our findings, we suggested potential areas of focus for quality improvement.

Further research on the relationship between health systems’ functions, performance and health outcomes in middle- and low-income countries with fragmented health systems is necessary to better understand their relationships and effects in different contexts.

## Declaration

Funding: International Development Research Centre (IDRC, Project No 103998) and the National Agency for Scientific and Technologic Promotion (ANPCyT, Project PICT No 1925/07).

Ethical approval: IDRC and ANPCyT.

Conflict of interest: none.
